# Supplementary figures and images for: 3D organoids containing endothelial and neural cells generation by serial inductions of differentiation on human iPSC-derived embryoid bodies
Source: bioRxiv. 2025 May 21:2025.05.20.653559. Preprint. [Version 1] doi: 10.1101/2025.05.20.653559 (PMC12139883; doi:10.1101/2025.05.20.653559)

SFig.1

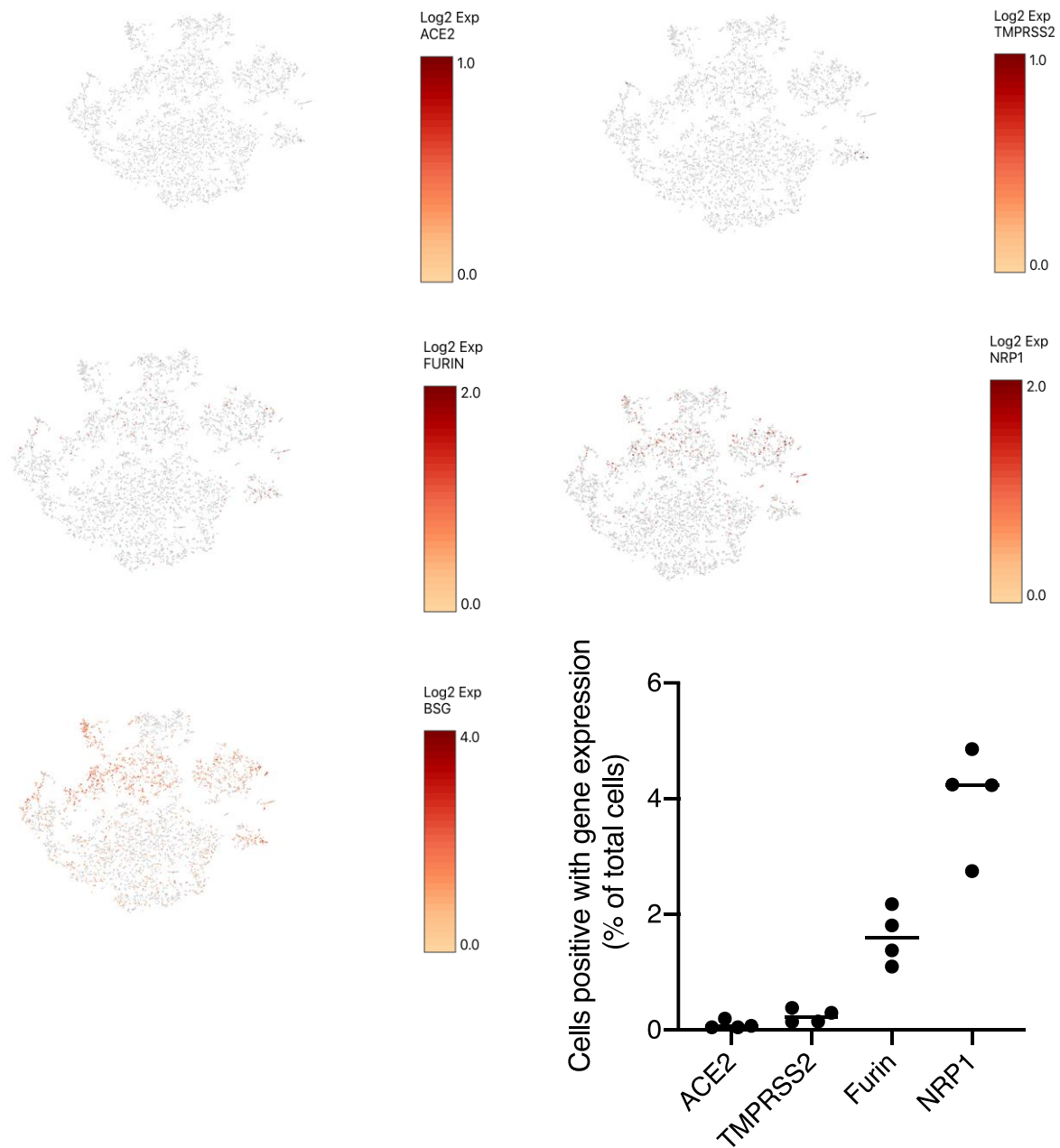

# A B SFig.2

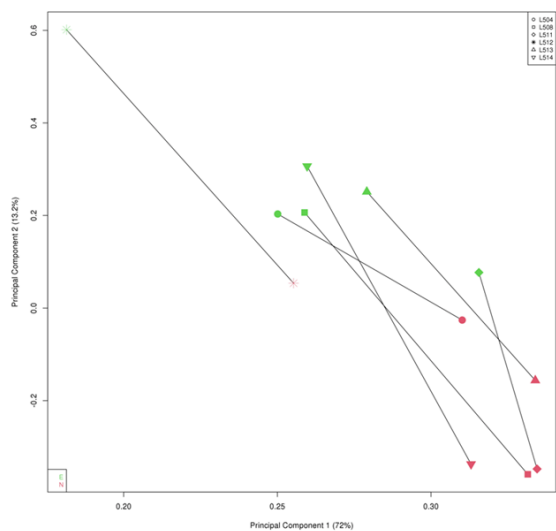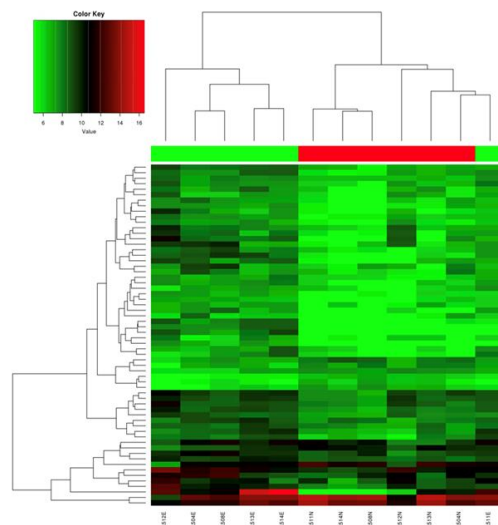

## C

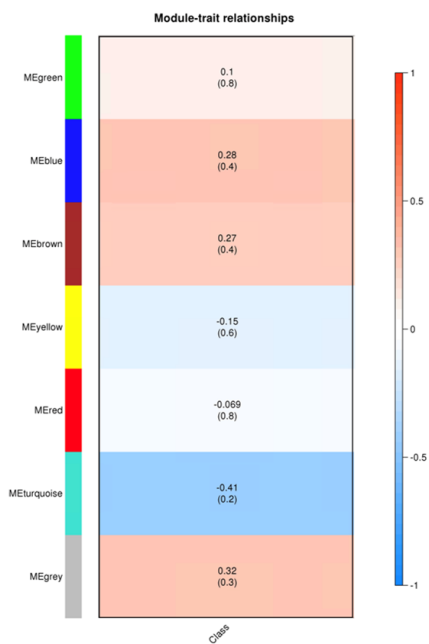

## D

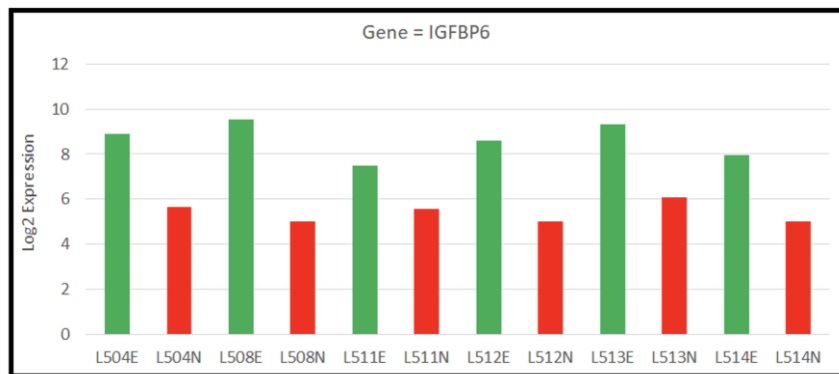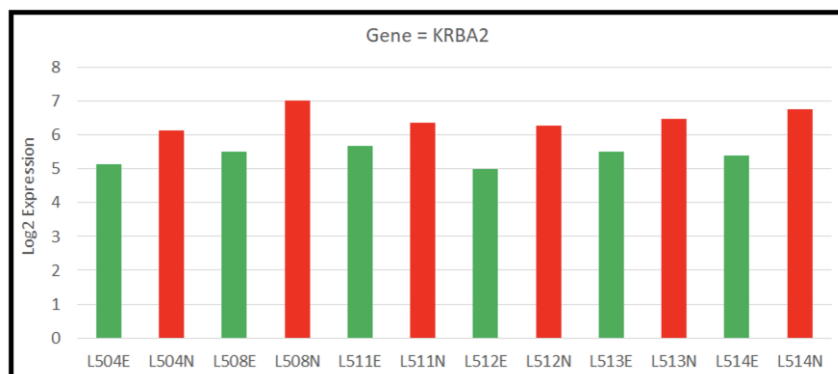

SFig.3

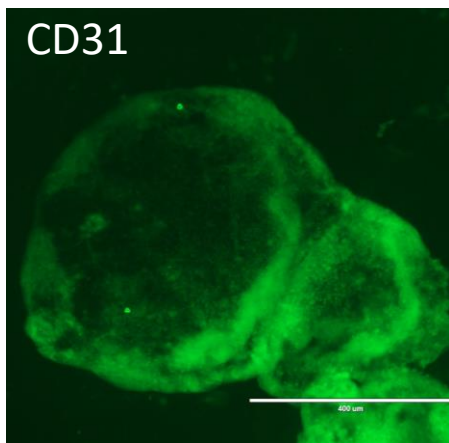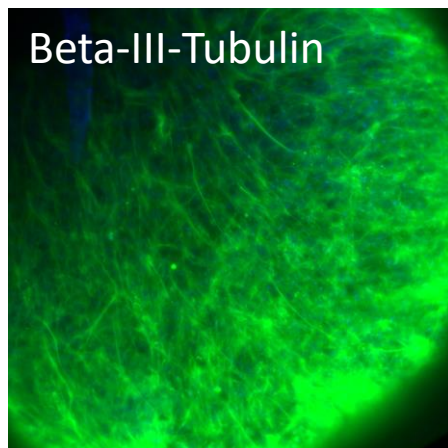

SFig. 4

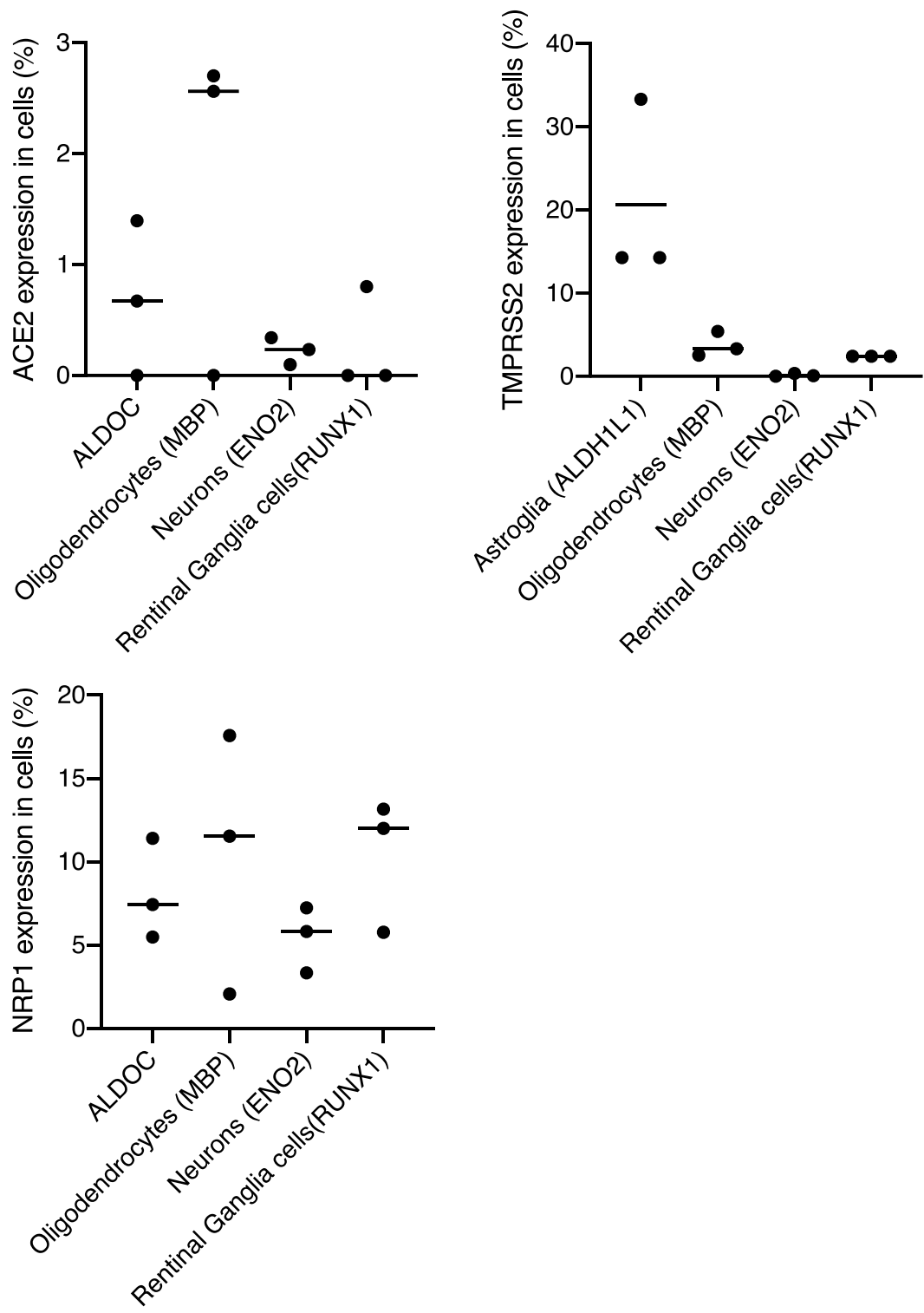

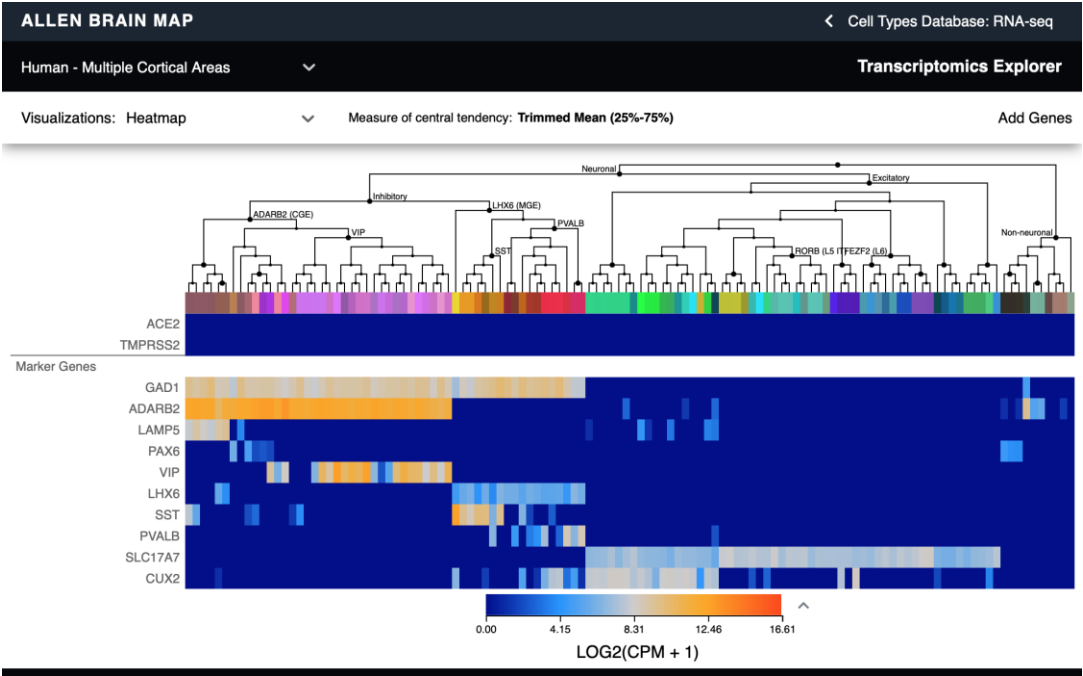

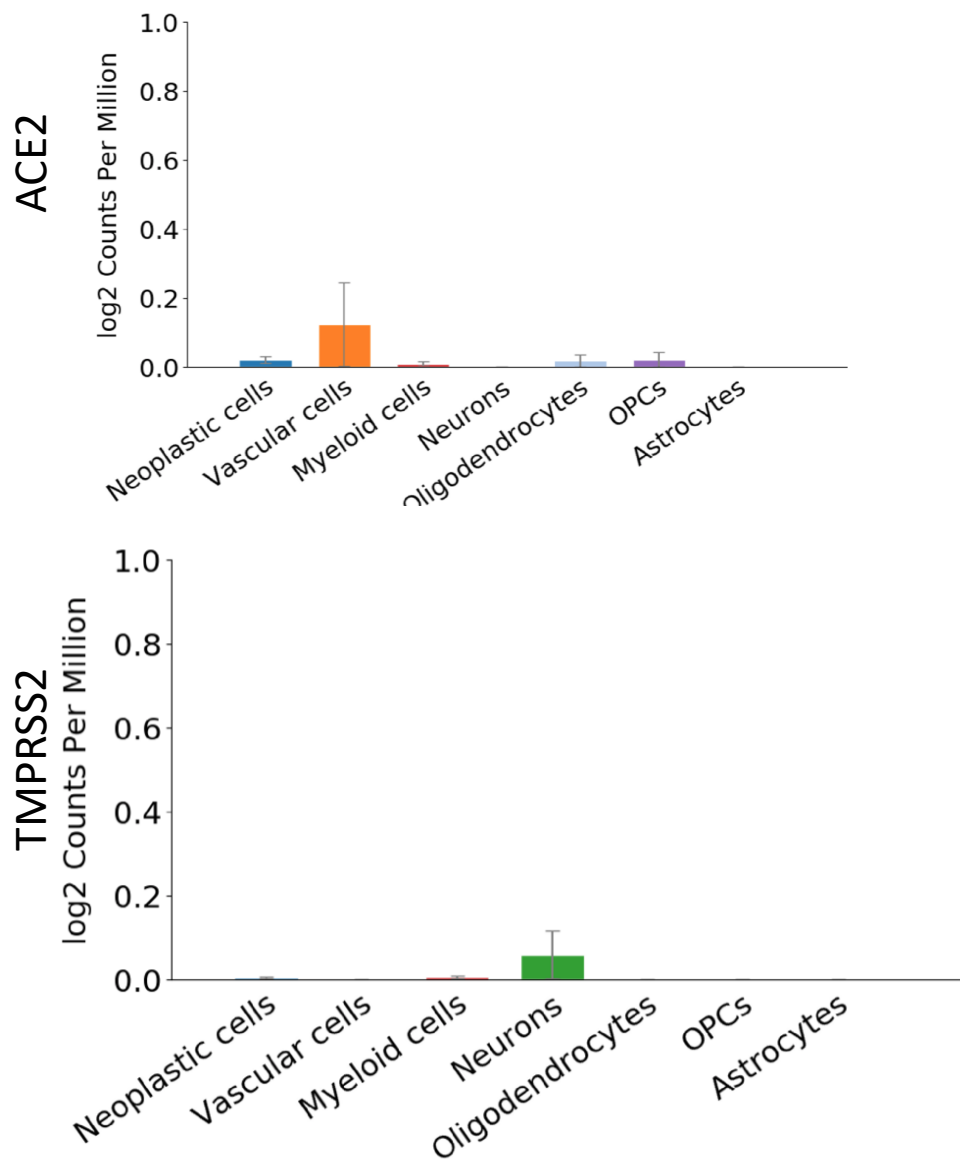

Supplement: Supplement 1 — SFig1. Expressions of SARS-CoV-2 binding associated proteins in 3D neuro-EC organoids. The cell distributions of ACE2, TMPRSS2, Furin and NRP1 in four organoids were detected by scRNA-Seq analysis. The data were presented as % of SARS-CoV-2 binding protein positive cells. SFig2. RNA-Seq analysis showed the IGFBP6 gene expression increased in EC-neural organoids (E) compared to cerebral organoids (N). While KRBA2 expression increased in cerebral organoids compared to EC-neural organoids. SFig3. Immunostaining of the organoids for endothelial cell marker CD31 and neuronal marker beat-III-tubulin. SFig4. SARS-CoV2 infection associated gene expression in the EC-neural organoids per cell types. SFig5. scRNA-Seq data from Allen Brain Atlas showed low levels of ACE2 and TMPRSS2 expression across brain cell types. SFig6. scRNA-Seq data from http://www.gbmseq.org/ showed low levels of ACE2 and TMPRSS2 expression in brain cells. [file media-1.pdf]
